# Supplementary material for: Patient-Reported Outcomes (PROs) and Patient Experiences in Fertility Preservation: A Systematic Review of the Literature on Adolescents and Young Adults (AYAs) with Cancer
Source: Cancers (Basel). 2023 Dec 13;15(24):5828. doi: 10.3390/cancers15245828 (PMC10741741; doi:10.3390/cancers15245828)
Supplement: Supplementary file 1 [file cancers-15-05828-s001.zip › cancers-2732235-supplementary.pdf]

Supplementary Materials: Patient-Reported Outcomes (PROs) and Patient Experiences in Fertility Preservation: A Systematic Review of the Literature on Adolescents and Young Adults (AYAs) with Cancer

Nicole F. Klijn, Moniek M. ter Kuile and Elisabeth E. L. O. Lashley

Table S1. Pubmed search selection.

**Pubmed search selection**
("fertility preservation"[MeSH] OR "fertility preservation"[tiab] OR "Fertility Preservations"[tiab] OR "Sperm Freezing"[tiab] OR ″Sperm banking″[tiab] OR "Egg Freezing"[tiab] OR "embryo banking"[tiab] OR "Oncofertility"[tiab] OR "assisted reproducti*"[tiab] OR "female infertility"[tiab] OR "Ovarian stimulation"[tiab] OR "embryo preservation"[tiab] OR "ovarian tissue cryopreservation"[tiab] OR (("Cryopreservation"[Mesh] OR "Cryopreservat*"[tiab]) AND ("ovar*"[tiab] OR "embryo*"[tiab] OR "oocyt*"[tiab] OR "sperm"[tiab] OR "testic*"[tiab]))) AND ("Patient Reported Outcome*"[tiab] OR "Questionnaire*"[tiab] OR "qualitative data"[tiab] OR "Patient-Reported Outcome*"[tiab] OR "Patient Outcome Assessment*"[tiab] OR "Patient-Centered Outcome*"[tiab] OR "Patient Centered Outcome*"[tiab] OR "Patient Outcomes Assessment*"[tiab] OR "Patient Outcome Assessment"[Mesh] OR "Patient Outcome Assessment*"[tiab] OR "Qualitative Research"[Mesh] OR "Qualitative Research"[tiab] OR "Surveys and Questionnaires"[Mesh] OR "Survey*"[tiab] OR ″communication″[tiab] OR "Respondent*"[tiab] OR "Nonrespondent*"[tiab] OR "Non-respondent*"[tiab] OR "psychological well-being"[tiab] OR "psychological wellbeing"[tiab] OR "patient-reported experience*"[tiab] OR "patient reported experience*"[tiab]) AND ("Neoplasms"[Mesh] OR "Neoplas*"[tw] OR "Tumor*"[tw] OR "Tumour*"[tw] OR "Cancer*"[tw] OR "malignan*"[tw] OR "oncolog*"[tw] OR "carcinoma*"[tw] OR "Medical Oncology"[Mesh])

Table S2. Quality assessment MMAT *.

**Qualitative studies**		1.1 Is the qualitative approach appropriate to answer the research question?	1.2. Are the qualitative data collection methods adequate to address the research question?	1.3. Are the findings adequately derived from the data?	1.4. Is the interpretation of results sufficiently substantiated by data?	1.5. Is there coherence between qualitative data sources, collection, analysis and interpretation?
1	Anazodo et al. [33]	Yes	Yes	Can't tell	Yes	Yes
2	Achille et al. [30]	Yes	Yes	Yes	Yes	Yes
3	Armuand et al. [34]	Yes	Yes	Yes	Yes	Yes
4	Bach et al. [13]	Yes	Yes	Yes	Yes	Yes
5	Benedict et al. [14]	Yes	Yes	Yes	Yes	Yes
6	Bentsen et al. 2021 [8]	Yes	No	Yes	Yes	Yes
7	Bentsen et al. 2023 [15]	Yes	Yes	Yes	Yes	Yes
8	Canzona et al. 2021 [35]	Yes	Yes	Yes	Yes	Yes
9	Canzona et al. 2023 [36]	Yes	Yes	Yes	Yes	Yes
10	Chapple et al. [31]	Yes	Yes	Can't tell	Yes	Yes
11	Cordeiro Mitchell et al. [16]	Yes	Yes	Yes	Can't tell	Can't tell
12	Corney et al. [17]	Yes	Yes	Can't tell	Can't tell	Yes
13	Crawshaw et al. [37]	Yes	Yes	Yes	Yes	Yes
14	Dahhan et al. [18]	Yes	Yes	Yes	Yes	Yes
15	Del Valle et al.[19]	Yes	Yes	Yes	Yes	Yes
16	Ehrbar et al. 2016[20]	Yes	Yes	Yes	Can't tell	Can't tell
17	Garvelink et al.[21]	Yes	Yes	Yes	Yes	Yes
18	Hershberger et al.[22]	Yes	Yes	Yes	Yes	Yes
19	Inhorn et al.[23]	Yes	Yes	Yes	Can't tell	Yes
20	Kirkman et al. [24]	Yes	Yes	Yes	Yes	Yes
21	Komatsu et al. 2014 [25]	Yes	Yes	Yes	Yes	Yes
22	Komatsu et al. 2018 [26]	Yes	Yes	Yes	Yes	Yes
23	Latif and al. [32]	Yes	Yes	Can't tell	Can't tell	Can't tell
24	Levin et al. [38]	Yes	Yes	Yes	Yes	Can't tell
25	Niemasik et al. [27]	Yes	Yes	Can't tell	Can't tell	Can't tell
26	Parton et al. [39]	Yes	Yes	Can't tell	Yes	Yes
27	Peddie et al. [9]	Yes	Yes	Yes	Yes	Yes
28	Salsman et al. [40]	Yes	Yes	Yes	Yes	Yes
29	Srikanthan et al [28]	Yes	Yes	Yes	Yes	Yes
30	Wang et al. [41]	Yes	Yes	Yes	Yes	Yes
31	Yee et al. 2012 [29]	Yes	Can't tell	Can't tell	Yes	Yes

**Quantitative studies**		4.1. Is the sampling strategy relevant to address the research question?	4.2. Is the sample representative of the target population?	4.3. Are the measurements appropriate?	4.4. Is the risk of nonresponse bias low?	4.5. Is the statistical analysis appropriate to answer the research question?
1	Bastings et al. [42]	Yes	Yes	Yes	Yes	Yes
2	Baysal et al.[43]	Yes	Can't tell	Yes/No	No	Yes
3	Edge et al. [58]	Yes	Yes	Can't tell	No	Yes
4	Hill et al. [44]	Can't tell	Can't tell	Yes	Yes	Yes
5	Kayiira et al. [66]	Yes	Can't tell	Can't tell	No	Yes
6	Ko et al. [45]	Yes	Yes	Can't tell	Yes	Yes
7	Krouwel et al. [59]	Yes	Can't tell	Yes	No	Yes
8	Leflon et al. [46]	Yes	Yes	Yes	Yes	Yes
9	Lewinsohn et al.[47]	Yes	Yes	Yes	Yes	Yes
10	Marino et al. [67]	Yes	Yes	Yes	Can't tell	Yes
11	Melo et al. [48]	Yes	Yes	Yes	Yes	Yes
12	Mersereau et al. [49]	Yes	Can't tell	Can't tell	Yes	Yes
13	Pacey et al. [60]	Yes	Yes	Yes	Yes	Yes

14	Perez et al. [61]	Yes	Yes	Yes	Yes	Yes
15	Ruddy et al. [50]	Yes	Yes	Yes	Yes	Yes
16	Ruggeri et al. [51]	Yes	Yes	Can't tell	Yes	Yes
17	Sauerbrun et al.[52]	Yes	Can't tell	Can't tell	No	Yes
18	Schover et al. [62]	Yes	Yes	Yes	No	Yes
19	Urech et al. [53]	Yes	Can't tell	Yes	Can't tell	Yes
20	Van den Berg et al.[54]	Yes	Can't tell	Yes	No	Yes
21	Van Wolff et al. [56]	Yes	Can't tell	Can't tell	Can't tell	Yes
22	Walasik et al. [55]	Yes	Can't tell	Can't tell	Can't tell	Yes
23	Xi et al. [63]	Yes	Yes	Yes	Yes	Yes
24	Yee et al. [64]	Yes	Yes	Yes	No	No
25	Zanagnolo et al. [57]	Yes	Can't tell	Yes	No	Yes
26	Zhang et al.[65]	Yes	Can't tell	Yes	Can't tell	Yes
**Mixed Methods studies**		5.1. Is there an adequate rationale for using a mixed methods design to address the research question?	5.2. Are the different components of the study effectively integrated to answer the research question?	5.3. Are the outputs of the integration of qualitative and quantitative components adequately interpreted?	5.4. Are divergences and inconsistencies between quantitative and qualitative results adequately addressed?	5.5. Do the different components of the study adhere to the quality criteria of each tradition of the methods involved?
1	Ehrbar et al. 2022 [70]	Yes	Yes	Yes	Can't tell	Yes
2	Schlossman et al. [68]	Yes	Yes	Yes	Can't tell	Yes
3	Ussher et al.[71]	Yes	No	Yes	No	Can't tell
4	Vogt et al.[69]	Yes	Yes	Yes	Yes	Yes

* In 85% of the articles the primary scoring was in accordance
